# Undercarboxylated osteocalcin as a biomarker of subclinical atherosclerosis in non-dialysis patients with chronic kidney disease

**DOI:** 10.1186/s12929-015-0183-6

**Published:** 2015-09-17

**Authors:** Minfang Zhang, Zhaohui Ni, Wenyan Zhou, Jiaqi Qian

**Affiliations:** Renal Division, Renji Hospital, School of Medicine, Shanghai Jiaotong University, 160 Pujian Road, Shanghai, 200127 China

**Keywords:** Osteocalcin, Atherosclerosis, Chronic kidney disease, Biomarker

## Abstract

**Background:**

Studies in recent years have shown that undercarboxylated osteocalcin (uOC) not only maintains bone mineralization, but is also involved in the regulation of atherosclerosis. However, a correlation between uOC and carotid atherosclerosis in non-dialysis patients with chronic kidney disease (CKD) has not been investigated.

A total of 240 non-dialysis patients with CKD were included in the study. For these patients, the median estimated glomerular filtration rate (eGFR) was 20.05 (12.43–49.32) ml/min/1.73m^2^. Serum uOC levels were measured using enzyme-linked immunosorbent assay (ELISA). Carotid ultrasonography was performed to assess carotid atherosclerotic plaques and intima–media thickness (IMT) in an attempt to analyze the relationship between uOC level and carotid atherosclerosis.

**Results:**

The uOC levels of non-dialysis patients with CKD were significantly lower than those of healthy controls [28.16 (21.40–45.85) ng/mL vs. 36.42 (28.05–49.28) ng/mL, *P* < 0.01]. The uOC levels gradually decreased as CKD progressed (*P* < 0.01). The uOC levels were significantly lower in patients with carotid plaques than in patients without carotid plaques [25.98 (20.14–31.35) ng/mL vs. 31.02 (25.86–36.40) ng/mL, *P* < 0.01]. uOC level showed significant negative correlation with IMT (*r* = -0.33, *P* < 0.01). Logistic regression analysis revealed that after adjustment for various confounding factors, decreased uOC levels were shown to indicate increased possibility of carotid atherosclerotic plaque development in non-dialysis patients with CKD (on every 1 SD decrease in the uOC level, odds ratio 1.70, 95 % confidence interval 1.24–2.98, *P* < 0.01). Multivariate stepwise regression analysis demonstrated that decreased uOC level (β = -0.163, P < 0.05) was an independent risk factor for increased carotid IMT in non-dialysis patients with CKD.

**Conclusion:**

Serum uOC levels in non-dialysis patients with CKD are significantly lower than those in healthy individuals, and uOC is closely associated with subclinical atherosclerosis in CKD patients.

## Background

The mortality rate of patients with chronic kidney disease (CKD) is much higher than that of an age-matched general population, with death caused by cardiovascular events accounting for over 50 % of deaths. Atherosclerotic cardiovascular disease (CVD) is considered the most common complication causing mortality in patients with CKD. Although the incidences of traditional risk factors are high in CKD patients, such as hypertension and diabetes, the high mortality rate of CVD in these patients cannot yet be fully explained [1–3].

Osteocalcin (OC) is a 49-amino acid, vitamin K-dependent calcium-binding protein which was secreted by mature osteoblasts. It is a specific biomarker of bone turnover and bone formation. OC plays a critical role in maintaining the bone mineralization rate and inhibiting hydroxyapatite crystallization and cartilage mineralization. Recent studies have shown that OC might also be involved in the regulation of energy, and glucose and lipid metabolism [4]. OC knockout mice demonstrated impaired glucose tolerance and clearly increased fat content as well as fat quantity. However, administration of recombinant OC promoted the proliferation of pancreatic ß cells, increased insulin level, enhanced energy metabolism, increased insulin sensitivity, and promoted adiponectin expression, suggesting an interaction between bone tissue and metabolism [5, 6]. In addition, multiple studies have demonstrated that OC is closely associated with the development and prognosis of atherosclerosis and CVD [7–14].

Circulating total osteocalcin (tOC) is comprised of two major forms of OC, undercarboxylated osteocalcin (uOC) and carboxylated osteocalcin (cOC). Recent studies on patients with hypertension and metabolic syndromes have shown that uOC is closely associated with the development of CVD [7, 8]. Alfadda et al. measured the levels of all three forms of OC in patients with type 2 diabetes, and found that uOC was a better predictor of CVD risk in these patients than tOC [15]. Animal and *in vitro* studies have shown that only the uOC elicits an endocrine effect, which maintains glucose homeostasis and regulates lipid metabolism [6]. The relationship between uOC levels and atherosclerosis in the CKD population has not been well investigated. Therefore, we performed a cross-sectional study to investigate the relationship between uOC levels and carotid atherosclerosis in non-dialysis patients with CKD.

## Methods

### Study subjects

Two hundred forty non-dialysis CKD patients (age, 50–75 years; men and postmenopausal women) admitted to our hospital were enrolled in this study. CKD is defined as either kidney damage or glomerular filtration rate (GFR) < 60 ml/min/1.73m^2^ for ≥ 3 months by the National Kidney Foundation. Kidney damage is manifested as pathologic abnormalities or markers of damage, including abnormalities in blood or urine tests or imaging studies. CKD patients can be divided into different stages according to the level of GFR: stage I (GFR ≥ 90 ml/min/1.73m^2^), stage II (GFR 60–89 ml/min/1.73m^2^), stage III (GFR 30–59 ml/min/1.73m^2^), stage IV (GFR 15–29 ml/min/1.73m^2^) and stage V (GFR < 15 ml/min/1.73m^2^). Exclusion criteria are as follows: (1) an acute cardio-vascular event (defined as acute coronary syndrome, new-onset cardiac arrhythmia, heart failure, cerebrovascular accident, peripheral vascular thrombosis, or arterial dissection) within one month; (2) infection or trauma within one month; (3) tumor; (4) familial hyperlipidemia; (5) acute exacerbation on CKD; (6) autoimmune disease; and (7) diabetes. Fifty age and sex-matched healthy individuals from the medical examination center were included as control group. The procedures used in this study met ethics requirements, and were approved by the ethics committee of Renji hospital. Informed consent forms were signed by all participants.

### Medical history collection

General characteristics of patients, including sex, age, smoking history, medication use, height, body weight, and body mass index (BMI) were recorded. Blood pressure was measured in the morning with the patient in a supine position after at least 10 min of rest using a sphygmomanometer with a cuff wrapped around the left upper arm positioned at the same level as the heart. The mean arterial blood pressure (MABP) was calculated according to the formula: MABP = diastolic blood pressure + 1/3 (systolic blood pressure − diastolic blood pressure).

### Laboratory assessment

Blood samples were collected after a 10-h fast. Hemoglobin, serum creatinine (Scr), blood urea nitrogen, uric acid, albumin, total cholesterol (TC), triglyceride (TG), high-density lipoprotein cholesterol (HDL-C), low-density lipoprotein cholesterol (LDL-C), fasting blood glucose (FBG), calcium, and phosphorus levels were measured using an automatic biochemistry analyzer (Bayer ADVIA 1650). High sensitivity C-reactive protein (hs-CRP) level was measured by rate nephelometry (Beckman Array 360 System, Deerfield, IL, USA). Estimated GFR (eGFR) was calculated using a MDRD-4 (Modification of Diet in Renal Disease Study) formula: 186 × (Scr/88.4)^-1.154^ × age^-0.203^ × (0.742 if female) [16].

### Analysis of serum uOC

Serum uOC level was measured using an enzyme-linked immunosorbent assay (ELISA) (Takara Bio Inc., Japan). The ELISA kit provides specific monoclonal antibodies reactive to uOC at amino acid positions 17, 21, and 24. The detection sensitivity was 0.25 ng/ml.

### Doppler ultrasonography of the carotid artery

Doppler ultrasonography of the carotid artery was performed by specialized professionals within one month of blood sampling using an HP-HX Color Doppler Ultrasound System (Hewlett-Packard Company, Palo Alto, CA, USA) with a 10 MHz transducer. The angle between the ultrasound beam and the direction of blood flow was <60°, and the intima-media thickness (IMT) was measured at 2 cm proximal to the bifurcation of the carotid artery. IMT was defined as the distance between the lumen-intima interface and the media-adventitia interface. An atherosclerotic plaque was defined as an echo-structure protruding into the vascular lumen with a thickness of >1 mm and more than twice the thickness of the neighboring sites [17]. Systolic peak velocity (SPV), diastolic peak velocity (DV), and resistance index (RI) were also determined.

### Statistical analysis

Quantitative variables were expressed as mean ± standard deviation or median (interquartile range). Two groups of normally distributed data were compared using an independent sample *t*-test, and the difference among multiple groups was analyzed using single-factor analysis of variance (ANOVA). A Mann–Whitney *U* test was performed to analyze the difference between two groups of non-normally distributed data, and the Kruskal–Wallis test was performed to compare the differences among multiple groups. The *χ*^2^ test was used to compare the differences in data constitution. Correlation analysis was performed using Pearson method. Multivariate logistic regression analysis was performed to investigate the relationship between uOC and carotid plaques. Multivariate stepwise linear regression analysis was performed to determine factors affecting carotid IMT. All statistical analyses were performed using SPSS software version 19.0, and *P* < 0.05 was considered statistically significant.

## Results

### General characteristics of non-dialysis patients with CKD

General characteristics of patients are presented in Table 1. The main etiology of CKD included the following: glomerulonephritis (*n* = 151, 62.92 %), hypertensive nephrosclerosis (*n* = 25, 10.42 %), tubulointerstitial nephritis (*n* = 7, 2.92 %), polycystic kidney disease (*n* = 2, 0.83 %), obstructive nephropathy (*n* = 2, 0.83 %), and CKD of unknown cause (*n* = 53, 22.08 %). There were significant differences in the levels of BMI, MABP, hemoglobin, eGFR, uric acid, TC, TG, LDL-C, HDL-C, phosphate and hs-CRP among different groups. As for the drug use, 97 (40.42 %) patients used angiotensin-converting enzyme inhibitor, 81 (33.75 %) patients used angiotensin receptor blocker, 135 (56.25 %) patients used calcium channel blocker, 62 (25.83 %) patients used β-blocker, 47 (19.58 %) patients used statin, 58 (24.17 %) patients used phosphate binder and 14 (5.83 %) patients used active Vitamin D.

**Table 1:** Baseline characteristics of CKD patients

	All CKD	CKD I-II	CKD III	CKD IV	CKD V	*P* value
N	240	41	75	58	66	
Age, year	61.83 ± 11.61	58.84 ± 12.44	60.96 ± 10.85	62.83 ± 11.41	63.82 ± 12.57	NS
Male, n (%)	151 (62.92 %)	30 (73.17 %)	54 (72.00 %)	33 (56.90 %)	34 (51.52 %)	< 0.01
BMI, kg/m^2^	23.68 ± 4.14	24.23 ± 4.35	24.64 ± 4.16	22.34 ± 3.54	23.26 ± 4.12	< 0.01
Smoke history, n (%)	49 (20.42 %)	6 (14.63 %)	14 (18.67 %)	16 (27.59 %)	13 (19.70 %)	NS
MABP, mmHg	100.67 (93.00–110.83)	95.33 (90.00–104.58)	96.67 (91.67–108.33)	103.33 (93.33–113.33)	103.67 (96.67–115.83)	< 0.05
FBG, mmol/L	5.03 ± 0.84	4.81 ± 0.62	5.26 ± 0.90	4.73 ± 0.75	5.15 ± 0.89	NS
Hemoglobin, g/dl	110.45 ± 31.26	135.79 ± 28.37	126.43 ± 26.54	108.58 ± 18.46	83.89 ± 18.45	< 0.01
Albumin, g/L	37.90 (33.25–40.50)	38.00 (32.10–42.50)	37.50 (35.00–41.50)	36.25 (32.50–39.50)	36.00 (32.05–39.10)	NS
eGFR, ml/min/1.73m^2^	20.05 (12.43–49.32)	83.57 (71.28–108.35)	44.56 (37.72–53.52)	24.53 (19.35–26.42)	8.17 (5.84–10.52)	< 0.01
Uric acid, mmol/L	467.51 ± 133.13	354.72 ± 114.87	445.16 ± 98.76	494.32 ± 112.53	509.58 ± 157.19	< 0.01
TC, mmol/L	4.74 (4.11–5.89)	4.83 (4.17–5.69)	5.15 (4.13–6.18)	5.31 (4.27–6.36)	4.36 (3.65–5.23)	< 0.01
TG, mmol/L	1.69 (1.22–2.52)	1.75 (1.12–2.59)	1.84 (1.26–2.87)	1.78 (1.36–2.51)	1.52 (1.12–2.16)	< 0.01
LDL-C, mmol/L	3.14 (2.40–3.97)	3.24 (2.58–4.07)	3.39 (2.57–4.31)	3.22 (2.71–4.14)	2.82 (2.31–3.49)	< 0.01
HDL-C, mmol/L	1.25 (1.02–1.49)	1.36 (1.13–1.64)	1.31 (1.00–1.58)	1.23 (1.12–1.53)	1.15 (1.03–1.56)	< 0.01
Calcium, mmol/L	2.18 ± 0.28	2.22 ± 0.34	2.15 ± 0.24	2.09 ± 0.31	2.14 ± 0.25	NS
Phosphate, mmol/L	1.50 ± 0.42	1.29 ± 0.37	1.35 ± 0.44	1.54 ± 0.50	1.71 ± 0.62	< 0.01
hs-CRP, mg/L	2.24 (1.00–4.10)	1.80 (1.00–3.50)	1.84 (1.00–4.87)	2.36 (1.00–3.56)	3.64(2.03–5.84)	< 0.01
*Drug use*						
ACEI, n (%)	97 (40.42 %)	30(73.17 %)	31 (41.33 %)	23 (39.66 %)	13 (19.70 %)	< 0.01
ARB, n (%)	81 (33.75 %)	17 (41.46 %)	28 (37.33 %)	19 (32.76 %)	17 (25.76 %)	NS
CCB, n (%)	135 (56.25 %)	10 (24.39 %)	38 (50.67 %)	32 (55.17 %)	55 (83.33 %)	< 0.01
β-blocker, n (%)	62 (25.83 %)	7 (17.07 %)	17 (22.67 %)	18 (31.03 %)	20 (30.30 %)	NS
Statin, n (%)	47 (19.58 %)	5 (12.20 %)	23 (30.67 %)	11 (18.97 %)	8 (12.12 %)	< 0.05
Phosphate binder, n (%)	58 (24.17 %)	0 (0 %)	7 (9.33 %)	13 (22.41 %)	38 (57.58 %)	< 0.01
Active VitD, n (%)	14 (5.83 %)	0 (0 %)	0 (0 %)	3 (5.17 %)	11 (16.67 %)	< 0.01

*BMI* body mass index, *MABP* mean arterial blood pressure, *FBG* fasting blood glucose, *eGFR* estimated glomerular filtration rate, *TC* total cholesterol, *TG* triglycerides, *LDL-C* low-density lipoprotein cholesterol, *HDL-C* high-density lipoprotein cholesterol, *hs-CRP* high-sensitivity C-reactive protein, *ACEI* angiotensin-converting enzyme inhibitor, *ARB* angiotensin receptor blocker, *CCB* calcium channel blocker, *VitD* vitamin D

### B-mode ultrasonography findings of the carotid artery in CKD patients

Carotid ultrasonography that was performed in all patients showed carotid plaques in 124 (51.67 %) patients. Carotid IMT gradually increased as eGFR decreased (*P* < 0.01). No significant differences in the ratio of carotid plaque formation and levels of SPV, DV and RI were observed among different groups of patients (Table 2).

**Table 2:** Carotid B-mode ultrasonography of CKD patients

	All CKD	CKD I-II	CKD III	CKD IV	CKD V	*P* value
N	240	41	75	58	66	
Carotid plaque, n (%)	124 (51.67 %)	18 (43.90 %)	36 (48.00 %)	32 (55.17 %)	38 (57.58 %)	NS
IMT (mm)	0.79 ± 0.23	0.66 ± 0.20	0.76 ± 0.29	0.83 ± 0.33	0.88 ± 0.30	< 0.01
SPV(cm/s)	78.49 ± 21.54	82.13 ± 18.05	79.96 ± 24.32	73.24 ± 17.78	79.75 ± 18.86	NS
DV(cm/s)	20.40 ± 8.12	25.23 ± 6.64	22.56 ± 8.41	22.14 ± 6.42	23.55 ± 7.24	NS
RI	0.72 ± 0.07	0.71 ± 0.11	0.72 ± 0.06	0.72 ± 0.06	0.74 ± 0.06	NS

*IMT* intima-media thickness, *SPV* systolic peak velocity, *DV* diastolic peak velocity, *RI* resistance index

### Significant decrease in uOC level in CKD patients

**Fig. 1:**
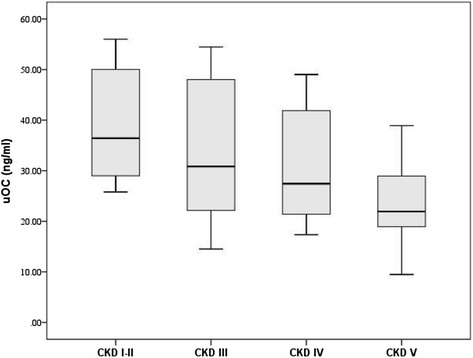
Relationship between uOC concentration and progression of CKD. uOC concentration decreased significantly with the progression of CKD (*P* < 0.01). This figure is a “box and whisker plot”. The center (box) represents the middle 50th percentile of the data set and is derived using the lower and upper quartile values. The median value (dark line) is displayed inside the box. The maximum and minimum values are displayed with vertical lines (whiskers) connecting the points to the center box

The uOC levels in non-dialysis CKD patients were significantly lower than those in healthy controls [28.16 (21.40–45.85) ng/mL vs. 36.42 (28.05–49.28) ng/mL, *P* < 0.01]. The uOC levels gradually decreased as CKD progressed (*P* < 0.01) (Fig. 1). Spearman’s correlation analysis demonstrated a significant positive correlation of the uOC level with eGFR (r = 0.48, *P* < 0.01) and HDL-C level (r = 0.43, *P* < 0.01) as well as a negative correlation with age (r = -0.34, *P* < 0.01), uric acid (r = -0.22, *P* < 0.05), and hs-CRP level (r = -0.36, *P* < 0.01) (Table 3).

**Table 3:** Correlations between uOC concentrations and clinical and echocardiographic parameters

Parameter	Spearman’s r	*P* value
Age, year	−0.34	< 0.01
BMI, kg/m^2^	0.10	NS
MABP, mmHg	0.04	NS
FBG, mmol/L	−0.22	NS
Hemoglobin, g/dl	0.10	NS
Albumin, g/L	0.05	NS
eGFR, ml/min/1.73m^2^	0.48	< 0.01
Uric acid, mmol/L	−0.22	< 0.05
TC, mmol/L	0.03	NS
TG, mmol/L	−0.12	NS
LDL-C, mmol/L	0.08	NS
HDL-C, mmol/L	0.43	< 0.01
Calcium, mmol/L	018	NS
Phosphate, mmol/L	0.01	NS
hs-CRP, mg/L	−0.36	< 0.01
IMT (mm)	−0.33	< 0.01
SPV(cm/s)	0.25	< 0.05
DV(cm/s)	0.20	< 0.05
RI	−0.24	< 0.01

*uOC* undercarboxylated osteocalcin, *BMI* body mass index, *MABP* mean arterial blood pressure, *FBG* fasting blood glucose, *eGFR* estimated glomerular filtration rate, *TC* total cholesterol, *TG* triglycerides, *LDL-C* low-density lipoprotein cholesterol, *HDL-C* high-density lipoprotein cholesterol, *hs-CRP* high-sensitivity C-reactive protein, *IMT* intima-media thickness, *SPV* systolic peak velocity, *DV* diastolic peak velocity, *RI* resistance index

### Correlation between uOC level and carotid plaque formation in CKD patients

The uOC levels were significantly lower in patients with carotid plaques than in patients without carotid plaques [25.98 (20.14–31.35) ng/mL vs. 31.02 (25.86–36.40) ng/mL, *P* < 0.01]. Logistic regression analysis showed that decreased uOC level indicated increased possibility of carotid plaque formation in non-dialysis CKD patients after adjustment for age, sex, BMI, smoking history, MABP, eGFR, therapeutic medication use, and FBG, TC, TG, LDL-C, HDL-C, and hs-CRP levels (every 1 SD decrease in uOC level, odds ratio 1.70, 95 % confidence interval 1.24–2.98, *P* < 0.01).

### Correlation between uOC level and carotid IMT in CKD patients

Univariate correlation analysis demonstrated that the uOC level had a significant negative correlation with IMT (r = -0.33, *P* < 0.01, Table 3). This correlation persisted after adjustment for age (r = -0.28, *P* < 0.01). Likewise, multivariate stepwise regression analysis showed that age (β = 0.289, *P* < 0.01), hs-CRP level (β = 0.146, *P* < 0.05), and uOC level (β = -0.163, *P* < 0.05) were independent risk factors for increased carotid IMT in non-dialysis CKD patients (Table 4). The original model included all variables significantly associated with IMT on univariate analysis, including age, BMI, hemoglobin, MABP, TC, TG, HDL-C, eGFR, uric acid, hs-CRP and uOC.

**Table 4:** Multivariate stepwise regression analysis for IMT

	Dependent variable:IMT	
	Standardized β	*P*
Age	0.289	< 0.01
lg (hs-CRP)	0.146	< 0.05
lg (uOC)	−0.163	< 0.05

Output from a multivariate stepwise regression model which showed that uOC was an independent marker for IMT

## Discussion

This cross-sectional study showed that serum uOC levels in non-dialysis CKD patients were significantly lower than those in healthy controls. The decrease in uOC level was closely associated with the development of carotid plaque, and was an independent risk factor for increased IMT in CKD patients. In the general population, previous study showed the negative correlation between uOC level and carotid atherosclerosis [18]. Our study was consistent with the results of general population, and expanded this association to the non-dialysis CKD patient population. To our knowledge, the present study is the first of its kind, and the results are valuable for clarifying risk stratification of CVD in CKD patients.

A study on Korean postmenopausal women demonstrated that tOC level was negatively correlated with BMI [19]. Pittas et al. also reported a negative correlation between tOC level and body fat content in a population over 65 year old [20]. However, a correlation between uOC level and BMI was not observed in this study, which is similar to the previous studies [15], suggesting that tOC and uOC might play different roles in energy metabolism. Dyslipidemia has been recognized as an independent risk factor for CVD. The main components of atherosclerotic plaque are large amounts of deposited cholesterol and cholesterol esters. Animal studies have shown that continuous subcutaneous infusion of recombinant OC significantly improved serum lipid level in wild type mice [6]. Our results demonstrated that serum uOC level was positively correlated with HDL-C level in non-dialysis CKD patients, indicating that uOC might participate in the regulation of lipid metabolism.

Studies on the correlation between OC level and CVD have yielded various results. OC level has been reported to show a significant negative correlation with brachial ankle pulse wave velocity and IMT in patients with type 2 diabetes, and has been speculated to function as a protective factor against CVD [9]. Another study reported that serum OC level was significantly lower in patients with coronary heart disease (CHD) than in patients without CHD, and that serum OC level was significantly inversely correlated with the extent of coronary stenosis [10]. In a young adult population, increased risk of CVD has been observed in patients with lower OC level [11]. In contrast to these results, increased OC level was shown to be associated with high incidence of carotid atherosclerosis in postmenopausal women and patients with type 2 diabetes [12–14]. The differences in these results can be explained by the varying degree of carboxylation of OC in the patients. A previous study showed that only the undercarboxylated part of serum OC was involved in the process of vascular calcification [21]. Recent studies suggested that uOC could serve as a novel biomarker for carotid atherosclerosis in patients with hypertension or metabolic syndrome [7, 8]. The present study also demonstrated that uOC level was lower in CKD patients with carotid plaques and that uOC level inversely correlated with IMT, indicating that uOC might play a critical role in preventing carotid atherosclerosis and CVD in non-dialysis CKD patients.

Numerous reports indicate that bone-associated proteins are involved in the process of atherosclerosis and calcification. Bini et al. studied human carotid endarterectomy specimens and showed that OC, osteopontin, and osteonectin were expressed both in large calcified areas as well as in small calcium deposits in atherosclerotic carotid arteries [22]. Gossl et al. studied endothelial progenitor cells (EPCs) in the circulating blood of patients with atherosclerosis, and found that EPCs expressed high levels of OC when they migrated to repair vascular injuries, and promoted arterial calcification at the same time [23]. Similar results were obtained from animal studies. A study on rapid aortic injury in rabbits induced by balloon angioplasty demonstrated that calcium deposits were found in necrotic areas 2–4 days after the injury and that OC expression was detected 14 days after vascular injury, indicating that OC was involved in the regulation of atherosclerosis development [24]. Matrix Gla Protein (MGP) and OC are well known Gla proteins. Rapid arterial calcification developed in MGP knockout mice, suggesting that MGP might be a key factor in the process of calcification [25]. OC and MGP display the same expression profile in humans. Therefore, OC and MGP, as Gla proteins, might play critical roles in both bone mineralization and vascular calcification. However, the underlying mechanisms by which OC regulates atherosclerosis and calcification need to be further studied.

Atherosclerosis is a chronic inflammatory process. CRP, which is an acute phase reactant produced by the liver, is not only a marker for CVD, but is also associated with the development and progression of atherosclerosis. The results of this study demonstrated that serum uOC level had a significant negative correlation with CRP level, indicating that the microinflammatory condition in CKD patients might be involved in the development of atherosclerosis. CRP not only regulates the aggregation of monocytes, but also simulates the production of tissue factors, activates complements and coagulation, thereby promoting formation and development of atherosclerotic plaques [26].

The limitations of this study are listed as follows. Firstly, the causality between the decreased uOC level and development of atherosclerosis could not be revealed through this observational study. Due to the cross-sectional design, the mechanism underlying the correlation between uOC level and carotid atherosclerosis could not be elucidated. Secondly, although numerous CVD risk factors such as hypertension and hyperlipidemia were strictly controlled, the possibility that underlying diseases and the corresponding medications interfered with the results cannot be ruled out. Finally, a prospective study with some clinical endpoints is required to clarify the role of uOC in the development of CVD in CKD patients.

## Conclusions

This study showed that decreased uOC levels were closely associated with carotid atherosclerosis in non-dialysis CKD patients. uOC may be not only a simple indicator of mineralization, but also an important factor that links bone metabolism and CVD. Further study is needed to determine if uOC could serve as a novel biomarker for predicting atherosclerosis and CVD in CKD patients.

## Abbreviations

CKD: Chronic kidney disease
CVD: Cardiovascular disease
OC: Osteocalcin
tOC: Total osteocalcin
uOC: Undercarboxylated osteocalcin
cOC: Carboxylated osteocalcin
BMI: Body mass index
MABP: Mean arterial blood pressure
Scr: Serum creatinine
TC: Total cholesterol
TG: Triglyceride
HDL-C: High-density lipoprotein cholesterol
LDL-C: Low-density lipoprotein cholesterol
FBG: Fasting blood glucose
hs-CRP: High sensitivity C-reactive protein
eGFR: Estimated glomerular filtration rate
MDRD: Modification of diet in renal disease
ELISA: Enzyme-linked immunosorbent assay
IMT: Intima-media thickness
ANOVA: Analysis of variance
CHD: Coronary heart disease
EPCs: Endothelial progenitor cells
MGP: Matrix Gla protein
ACEI: Angiotensin-converting enzyme inhibitor
ARB: Angiotensin receptor blocker
CCB: Calcium channel blocker
VitD: Vitamin D
SPV: Systolic peak velocity
DV: Diastolic peak velocity
RI: Resistance index
